# A guide to sharing open healthcare data under the General Data Protection Regulation

**DOI:** 10.1038/s41597-023-02256-2

**Published:** 2023-06-24

**Authors:** Jip W. T. M. de Kok, Miguel Á. Armengol de la Hoz, Ymke de Jong, Véronique Brokke, Paul W. G. Elbers, Patrick Thoral, Alejandro Castillejo, Tomás Trenor, Jose M. Castellano, Alberto E. Bronchalo, Tobias M. Merz, Martin Faltys, Collaborator group, Cristina Casares, Araceli Jiménez, Jaime Requejo, Sonia Gutiérrez, David Curto, Gunnar Rätsch, Jan M. Peppink, Ronald H. Driessen, Eric J. G. Sijbrands, Erwin J. O. Kompanje, Armand R. J. Girbes, Jose Barberan, Jose Felipe Varona, Paula Villares, Iwan C. C. van der Horst, Minnan Xu, Leo Anthony Celi, Bas C. T. van Bussel, Xavier Borrat

**Affiliations:** 1 Department of Intensive Care Medicine, Maastricht University Medical Centre+, Maastricht, the Netherlands; 2 Cardiovascular Research Institute Maastricht (CARIM), Maastricht University, Maastricht, the Netherlands; 3 Big Data Department, PMC, Fundacion Progreso y Salud (FPS), Regional Ministry of Health of Andalucia, Seville, Andalucia Spain; 4 Philips, Eindhoven, the Netherlands; 5 Center for Critical Care Computational Intelligence (C4I), Department of Intensive Care Medicine, Amsterdam University Medical Centers, Vrije Universiteit, Amsterdam, The Netherlands; 6 Sanitas - Data4Good, Madrid, Spain; 7 Fundación de Investigación HM Hospitales, Grupo HM Hospitales, Madrid, Spain; 8 Cardiovascular Intensive Care Unit, Auckland City Hospital, Auckland, New Zealand; 9 Department of Intensive Care Medicine, University Hospital, University of Bern, Bern, Switzerland; 10 Philips Research North America, Cambridge, MA USA; 11 Laboratory for Computational Physiology, Harvard-MIT Division of Health Sciences & Technology, Cambridge, Massachusetts, USA; 12 Department of Medicine, Beth Israel Deaconess Medical Center, Harvard Medical School, Boston, Massachusetts USA; 13 Department of Biostatistics Harvard T.H, Chan School of Public Health, Boston, Massachusetts USA; 14 Care and Public Health Research Institute (CAPHRI), Maastricht University, Maastricht, the Netherlands; 15 Anaesthesiology and Critical Care Department, Hospital Clinic de Barcelona, Barcelona, Spain; 16 Medical Informatics Department, Hospital Clinic de Barcelona, Barcelona, Spain; 17 Sanitas Seguros, Madrid, Spain; 18 Sanitas Hospitales, Madrid, Spain; 19 Sanitas Mayores, Madrid, Spain; 20 Department of Computer Science, ETH Zürich, Zürich, Switzerland; 21 Department of Internal Medicine, Erasmus MC, Rotterdam, The Netherlands; 22 Department of Intensive Care Medicine, Erasmus MC, Rotterdam, The Netherlands; 23 Department of Internal Medicine, Hospital Universitario Monteprincipe, Grupo HM Hospitales, Madrid, Spain; 24 Department of Internal Medicine, Hospital Universitario Sanchinarro, Grupo HM Hospitales, Madrid, Spain

**Keywords:** Databases, Law, Policy

## Abstract

Sharing healthcare data is increasingly essential for developing data-driven improvements in patient care at the Intensive Care Unit (ICU). However, it is also very challenging under the strict privacy legislation of the European Union (EU). Therefore, we explored four successful open ICU healthcare databases to determine how open healthcare data can be shared appropriately in the EU. A questionnaire was constructed based on the Delphi method. Then, follow-up questions were discussed with experts from the four databases. These experts encountered similar challenges and regarded ethical and legal aspects to be the most challenging. Based on the approaches of the databases, expert opinion, and literature research, we outline four distinct approaches to openly sharing healthcare data, each with varying implications regarding data security, ease of use, sustainability, and implementability. Ultimately, we formulate seven recommendations for sharing open healthcare data to guide future initiatives in sharing open healthcare data to improve patient care and advance healthcare.

## Introduction

Healthcare data are increasingly essential for improving patient care, particularly in the Intensive Care Unit (ICU)^1–3^. In addition, the COVID-19 pandemic has increased the urgency to share cross-border healthcare data for public goals further^4–6^. Such data foster the development of models that could inform and assist healthcare providers with diagnoses and prognoses. However, the broader implementation of these models is hampered by the fact that models perform well in the development setting but often have relatively poor generalisability^7^ as data from only a few countries, using relatively homogenous patient populations, are used^8^. Furthermore, patient populations and care change continuously, reducing the efficacy of data-driven models over time^9^. Together, this can reduce model performance in specific settings and result in discrimination between patients based on their demographic traits, such as race^10^.

The apparent issue of models being based on homogenous data containing only specific patient populations can only be resolved by increasing available data from multiple sources such that all patient types can be included and evaluated during the modelling process. To this end, large open data sets are essential, meaning open to those who will use them appropriately, not anyone. The biggest concerns regarding data sharing are the involved privacy risks. However, when data are housed in a relatively open network environment, it becomes a very low-value target for those attempting to profit from the malpractice of data stealing^11^. Furthermore, it can seriously stimulate research and data democratisation. Nevertheless, sharing health data can be cumbersome and requires a team effort that combines expert knowledge of different disciplines, including ethics, privacy, healthcare, data infrastructure, and data science. One of the biggest challenges of sharing health data are the privacy concerns regarding the sensitivity of the data in question. In Europe, the General Data Protection Regulation (GDPR) is implemented to protect individuals’ data and privacy. Consequently, all European databases must adhere to the rules of the GDPR, which is extensive and complicated for laypeople, comprising of 173 recitals and 99 articles. However, it is a misconception that the GDPR counteracts health data sharing. On the contrary, the GDPR is designed as a guideline for safely sharing sensitive data, although it can be challenging to navigate due to its complexity, especially for healthcare data sharing^12^.

To illustrate how personal health data can be shared appropriately under the GDPR, this paper presents a survey of four successfully implemented open European databases containing Electronic Health Record (EHR) data of patients from the ICU. In addition, this study aims to establish the necessary steps and formulates several recommendations for creating future scientific open ICU databases based on the experiences and lessons learned from the previous success stories.

## Results

### Database content

Each of the four European databases contains ICU data, although HM Hospitales COVID-19 v4 (HM) also contains data from the emergency department. The AmsterdamUMCdb and HiRID databases are the largest, with over 23,000 and 33,000 admissions, respectively. They contain comprehensive (e.g., highly granular and serial data) and a diverse collection of variables, including patient demographics, physiology, diagnoses, treatments, and even imaging data such as X-rays in the case of HM. SANITAS Data4Good (2,056 patients) and HM (4,479 patients) are not as large and generic in content as the other two databases, as they were explicitly created for COVID-19 research (Table 1).

**Table 1:** Interview results summary table.

	AmsterdamUMCdb	HiRID	Sanitas Data4Good	HM Hospitales
Link	https://amsterdammedicaldatascience.nl/amsterdamumcdb/	https://physionet.org/content/hirid/1.1.1/	https://landing.sanitasweb.es/data/opendatacovid/english.html	https://www.hmhospitales.com/coronavirus/covid-data-save-lives/english-version
Country	Netherlands	Switzerland	Spain	Spain
Patient population	ICU and high dependency unit, all comers	ICU	Hospitalised COVID-19 patients	Hospitalised COVID-19 patients
Size	23,106 admissions of 20,109 patients	33,905 admissions 33,905 patients	2,056 patients	4,479 patients
Years	2003 ~ 2016	2008 ~ 2016	2020 ~ 2021	2020 ~ 2021
Data types	Vital signs, labs, medication	Vital signs, labs, medication, demographics	Vital signs, labs, medication, demographics	Vital signs, labs, medication, demographics, and images in DICOM format
Privacy method data sharing	Anonymised GDPR not applicable	Anonymised GDPR not applicable	Pseudonymised GDPR applicable	Anonymised GDPR not applicable
Consent	Not applicable	Yes	Not applicable	Not applicable
Re-use requirements	Research purposes, publish results, a collaboration with practising intensivist	Results of research using HiRID data must be published in the public domain	Research purposes	Research purposes, Publish results
Data access requirements	Training & signed license agreement	Training & signed license agreement	IRB & signed request form	IRB & signed request form
Standardisation and coding	SNOMED	SNOMED	ICD-10	ICD-10 & ATC
Data sharing portal	DANS EASY repository	Physionet	Local site	Local site
Team composition disciplines	1) Clinical care 2) Data engineering	1) Data science 2) Clinical care 3) Three PhD students	1) Data science 2) Legal & privacy 3) Clinical research 4) Communication 5) Public Affairs 6) Hospital and Insurance Business Units	1) Legal 2) IT 3) Clinical research
Size of investment	4 person-month	8 person-month	4 person-month	4 person-month
External advisory	Privacy & Ethical	Government agency	Law firm	None

This table contains a summary of the results from the questionnaires. Columns represent the four different databases, and the rows summarise the topics questioned. Size of the investment is indicated in terms of time (with persons required to work for a whole month as one).

**ICU**: Intensive Care Unit, **GDPR**: General Data Protection Regulation, **IRB**: Institutional Review Board, **COVID-19**: Coronavirus Disease 2019, **DICOM**: Digital Imaging and Communications in Medicine, **SNOWMED**: Systematised Nomenclature of Medicine Clinical Terms, **IT**: Information Technology, **ICD-10**: International Classification of Diseases 10th revision, **ATC**: Anatomical Therapeutic Chemical code, **DANS**: Data Archiving and Networked Services.

### Main objective of the database

The main objective for all databases was stimulating open science to benefit society and improve patient care. AmsterdamUMCdb is supported by the European Society of Intensive Care Medicine (ESICM) Data Sharing Initiative and HiRID by the Swiss Federal Institute of Technology (ETH) Zürich, supporting proper use of open data and the development of machine learning algorithms in healthcare. SANITAS and HM were published, among other reasons, to accelerate international research on COVID-19 (Table S1).

### Funding

All four open databases were funded predominantly by scientific institute grants. None of the databases had business models for open data, and the data are accessible free of charge via an individual access policy (Table S1). One team was dedicated to building the databases per organisation. These teams comprised 4–6 members from different disciplines, who expressed that time was their biggest investment, working many out-of-office hours.

### Resources

The publication of an open health database requires multidisciplinary team science. This involves physicians understanding intensive care medicine, IT specialists to manage the database infrastructure, communications departments involved in the communication strategy, both internal privacy, ethical and legal experts for advice, as well as external experts for complete data protection impact analyses (DPIA) and audit of the de-identification and governance strategies. Investment of time from the organisation and team, as well as financial funding, was expressed in working hours, corresponding to an estimated four full person-months for all databases, except HiRID, with eight person-months (Table 1).

### Legal

HiRID gave every patient, or their representative, the option to opt out of their data being used for research, thus excluding any individuals who refused to provide consent from the database. For AmsterdamUMCdb, data was collected and processed by relying on scientific research exemption (Art. 9(j) GDPR). The alternative, relying on consent, was deemed infeasible due to the large sample size. SANITAS and HM had a similar approach, although their sample size was smaller. AmsterdamUMCdb and SANITAS consulted in-house lawyers and external EU privacy and data protection experts who advised on the legal aspects. Independent legal experts, including experts specialized in privacy and data protection, audited the de-identification and governance strategies, and concluded that the data were adequately anonymised for public access (Table 1 & Table S1).

### Data de-identification strategy

SANITAS pseudonymised the dataset, removing direct identifiers, completed a Data Protection Impact Assessment (DPIA) and submitted it to an independent full data audit. The other three databases anonymised their data. HiRID performed k-anonymisation^13^ (see Supplementary K-anonymisation section^14^), a risk-based de-identification strategy, without an external audit. AmsterdamUMCdb had a similar approach, but iteratively, repeating the process until an external review committee representing privacy, data protection and ethics experts, patient organisations and supporting hospitals, was satisfied with the estimated re-identification risk. They assessed re-identification risk based on three hypothetical attack types, essentially making it a form of adversarial modelling^15^. More specifically, AmsterdamUMCdb conducted a DPIA, excluded patients who opted out (as is legally required), masked patient and admission IDs, deleted all direct identifiers, removed images and free text (except for laboratory variables), and made all dates relative to the admission date. HiRID removed all eighteen identifying data elements listed in HIPAA. Dates were shuffled randomly, ranging from 2100 to 2200, while preserving seasonality, time, and weekdays. Furthermore, patient age, height, and weight were binned into five categories. Measurements and medication with units that change over time were standardised to their latest unit, and free text was removed (Table 1 & Table S1).

### Ethical

HiRID and AmsterdamUMCdb have generic institutional review board (IRB) approval, and HM requires an applicant’s own IRB approval on top of their database IRB approval. In contrast, SANITAS has no IRB approval; therefore, researchers who use the dataset must apply for their own IRB. Nevertheless, the ethics committees’ main concern was securing patients’ privacy. Therefore, AmsterdamUMCdb consulted an ethics specialist for an independent ethics review. This external ethics review concluded that with adequate de-identification, the risk of patient re-identification was minimised to the extent that the benefits for patients in general far outweighed the re-identification risk.

### Governance

Each database implemented an obligatory data request form, through which a steering committee assesses both the requestor and research proposal, preventing data misuse. Request forms vary between databases, covering good clinical practice certification and signed scientific integrity statements, database-specific and general training courses, research protocol evaluation, mandatory inclusion of practising intensivists who govern the open database, audits by patient organisations, as well as privacy and ethical consultants, mandatory open code sharing for reproducibility purposes, intellectual property disclosure, and trust in applicants with a solid track record who investigates plausible objectives. The requirements vary per database, with only partial overlap between databases. AmsterdamUMCdb and HiRID require the applicant to fulfil a training course, whereas SANITAS and HM do not. In addition, the latter two require the applicant’s IRB approval, which is not required by AmsterdamUMCdb and HiRID, as these meet generic IRB approval. At SANITAS, data can be downloaded using a URL, which expires after five days (Table S1).

### Sustainable strategy

The four databases vary in their strategy for database updating (adding new samples and/or variables). HM has performed regular updates, including new patients. SANITAS did one manual update adding new cases. Currently, SANITAS prepares enrichment with clinical data from nursing homes. HiRID plans to grow its dataset by adding three years of patient data. AmsterdamUMCdb plans to grow and enrich the database, incorporating comorbidities and data from the microbiology department. However, this increases the re-identification risk, which could be overcome through database growth by including additional centres.

### Standardisation

All databases used some form of standardised terminology. For example, AmsterdamUMCdb uses the SNOMED (Systematized Nomenclature of Medicine Clinical Terms) nomenclature, and HiRID provides mapping from their terminology to SNOMED codes to maintain its original structure. SANITAS and HM use the ICD (International Classification of Diseases) 10 nomenclature, and the latter also uses ATC (Anatomical Therapeutic Chemical) coding for its drug items.

### Database impact

The four databases combined had over 500 data requests. The number of publications associated with these databases is growing and being cited more often. More specifically, since the opening of HiRID, there have been 200 requests per year. The original publication has 84 citations (by January 2022). HM has received roughly 180 requests, 95% approved. Data from the four databases have been used in hackathons, contributing to a data science learning environment and multidisciplinary mutual data understanding. AmsterdamUMCdb and HiRID use GitHub to share code and documentation, contributing to reproducibility (Table S1).

### Data modules, data exchange and documents

SANITAS’ and HM’s approach to sharing the data involves their dedicated websites. They require the requestor to fill in a request form, and data can be downloaded via an IP-specific URL, valid for 5 days if the request is approved. AmsterdamUMCdb and HiRID use external platforms. HiRID uses the Physionet platform^16^ to share the data and stores additional documentation on its website. Furthermore, HiRID has a GitHub repository with some example Python code illustrating how to load the data. AmsterdamUMCdb has a similar setup, except data access is provided via the EASY repository from the Dutch Archiving and Networked Services. GitHub is used to share documentation, for example, python code and package, which provides basic functionality for working with the database (Table S1).

## Discussion

### Summary

Many experiences and lessons have been learned from the accomplishments of the four open European ICU databases reviewed in this study. By comparing all four databases and identifying similarities and differences, the recommendations in Fig. 4 were formulated for publishing open European ICU databases. We found that all four databases had to account for similar challenges of sharing open health data. The most significant identified challenges were the ethical and legal aspects due to the sensitivity of healthcare data, forcing these open data projects to abide by the applicable privacy and data protection laws and regulations. Consequently, data anonymisation and pseudonymisation were performed, although each database implemented this differently. In most cases, data was not collected on basis of explicit consent. We also found that IRB approval is essential for sharing open health data, but this was also addressed differently. AmsterdamUMCdb, HiRID, and HM acquired general IRB approval, while SANITAS did not. However, SANITAS and HM require IRB approval per research question accompanying a data request. Although stricter governance such as required IRB approval can make the data less easily accessible, it can reduce the risks of data misuse. Following the FAIR principles for Scientific Data Management (Findable, Accessible, Interoperable, and Reusable), the data should be Findable and Accessible^17,18^. Therefore, the data, metadata, and possibly additional content, like coding templates, should be easily accessible. To achieve findability and accessibility, AmsterdamUMCdb and HiRID chose to use existing data-sharing platforms. In contrast, SANITAS and HM share dedicated download links, a cheaper solution requiring more manual labour. We believe that all code used for analizing the data should be made publicly available to anyone, as this allows for replication and validation of previous research, while allowing other researchers to improve upon previous work^19^. The FAIR principles also state that data should be interoperable, for which standardised terminology is a crucial aspect^17^. Since all databases used standardised data formats, they all satisfied this criterion for interoperability. Harmonisation between open ICU healthcare databases is also important to improve interoperability, for example, to facilitate collaboration between European and US open data projects.

### Rising popularity of sharing open data

Open data is gaining attention. For example, the US National Institute of Health (NIH) issued in early 2022 that starting from 2023, all NIH-funded projects must include an annual ‘data management and sharing plan’ in their grant applications and make their data publicly available^20^. Also, the Dutch ZonMw promotes open science and FAIRification of health research^21^, and Stanford’s AI Medicine and Imaging is expanding its free^22^ repository of datasets for researchers worldwide. In Europe, the Data Governance Act (DGA)^23^ promotes data sharing and aims to build a trustworthy environment, facilitating novel research and the production of innovative products and services. One of the critical pillars of the DGA fosters data altruism across the EU by making it easier for individuals and companies to voluntarily make their data available for the common good, such as medical research projects. However, EU laws on processing (personal) data, such as the GDPR^24^, remain a challenging factor when sharing personal health data, as this means more stringent data protection rules are applicable. In the United States, comparable laws are written in the Health Insurance Portability and Accountability Act (HIPAA)^25^ to provide clarity and safety when handling healthcare data. The primary difference between the GDPR and HIPAA is that the GDPR’s purpose is the protection of personal data, which is considered a human right as per the EU Charter of Fundamental Rights, whereas HIPAA originated from insurance laws (more elaborately described in the supplementary information^14^). Such differences in legislation complicate international data sharing^26^. The GDPR has some stricter rules compared to HIPAA, making it unattractive for US instances to incorporate EU data in their public data repositories. Consequently, EU citizens are insufficiently represented in research on open data^27^. Therefore, we argue that European institutes should openly share patient data, such that European patients are incorporated in developing novel health technologies and can benefit from them accordingly.

### Data protection strategies

**Fig. 1:**
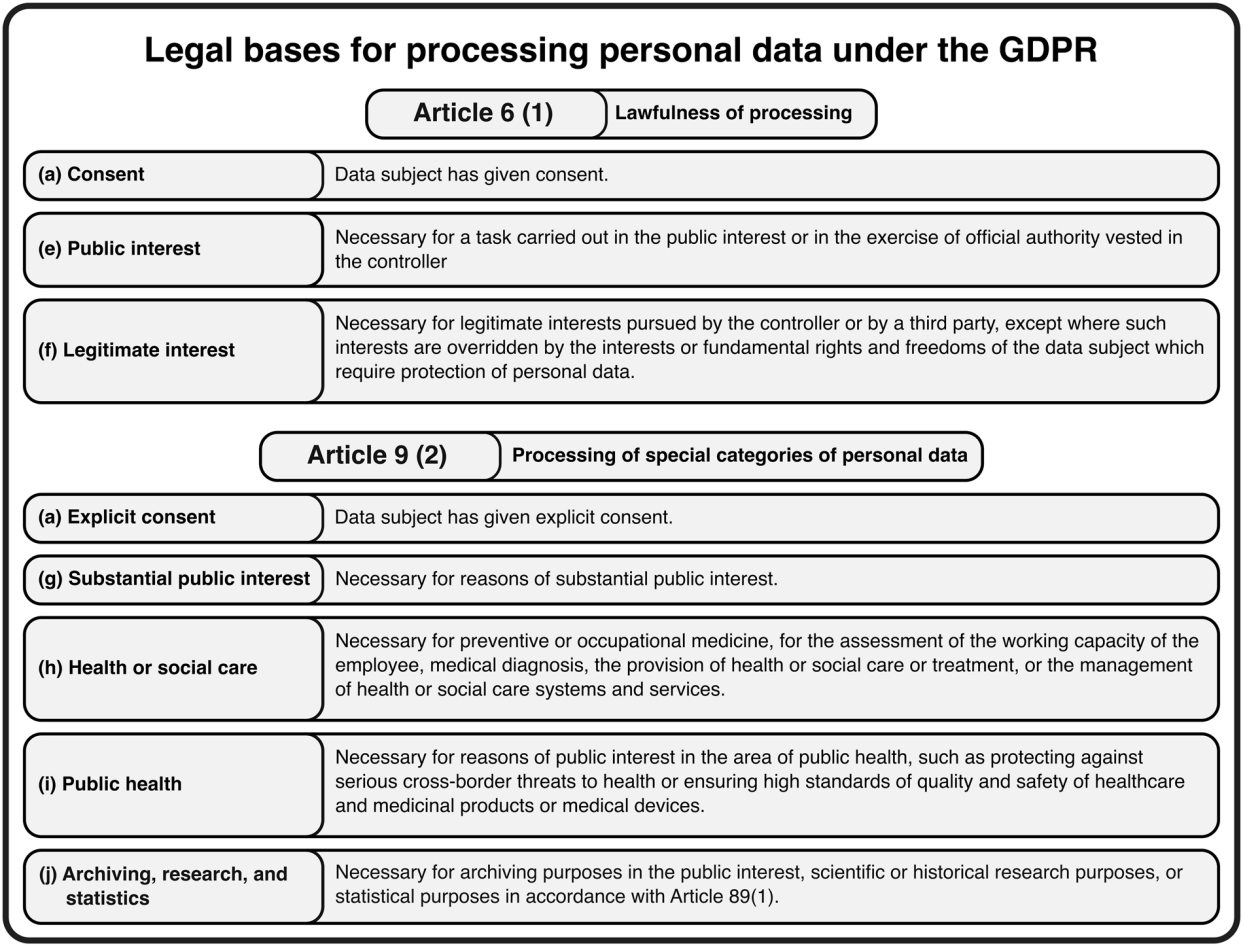
Legal bases for processing ‘special category’ personal data relevant to observational retrospective health research purposes from GDPR Article 9 (2). Implementation of the legal bases can differ between European countries. More elaborate descriptions of the legal bases and their implementations can be found in the Supplementary section on Pseudonymisation – GDPR applicable^14^.

Processing of pseudonymised data falls within the scope of the GDPR as pseudonymised data still qualifies as personal data, which may not be processed unless it falls under the legal bases described in Article 6 (1) and exceptions in Article 9 (2) of the GDPR^24^ (Fig. 1) (see supplementary information^14^). Alternatively, anonymising personal data can also allow for processing such data, although a valid legal basis and exception is still mandatory for the anonymisation process itself, as it is still personal data prior to anonymisation. Nevertheless, the difficulty in the GDPR framework is that, unlike in HIPAA, there is no concrete list of requirements for data to be considered de-identified. The definitions are dynamic and contextual, and there is a lack of clarity on key data protection concepts and requirements, resulting in inconsistencies in how anonymisation is viewed across EU Member States. Nevertheless, since the general doctrine is to strive for maximum effort in anonymisation, one of the strategies followed has been to assess the risk of re-identification, as was done by AmsterdamUMCdb and HiRID. Both performed k-anonymisation (see K-anonymisation Supplementary section^14^). SANITAS and HM delegated the assessment of re-identification to an external entity. As a suggestion for future databases, an additional approach to strengthen security is cloud computing with a safety layer. This entails that, rather than allowing the user to download the data, they are given access to a virtual environment where they can analyse it. This reduces the risk of sharing data without permission or stealing from a user. Furthermore, since all analyses are run in an online environment, this allows for query monitoring. Auditing queries in the virtual environment could detect nefarious efforts such as patient re-identification. The EU aims to develop a suitable European cloud environment for such use cases^28,29^.

### Four approaches to sharing healthcare data

**Fig. 2:**
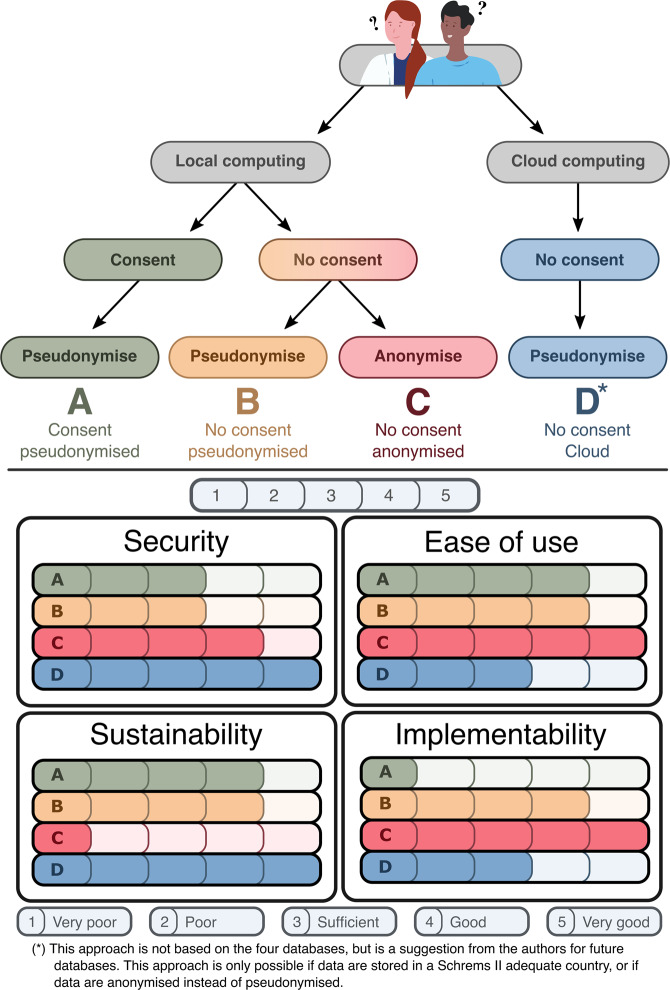
Diagram illustrating the four approaches to publishing an open ICU database. Based on the four successful open ICU databases and rules ordained in the GDPR, we present four possible approaches for openly sharing sensitive healthcare data. These are not the only possible options for sharing healthcare data, but the approaches we believe to be most common or appropriate. The arrows indicate different choices to be made and what combinations of those are possible, each ending up in a different terminal node A, B, C, or D; the four approaches for publishing an open ICU database. The first node depicts whether the data can be processed locally or in the cloud, and the second node shows whether data was shared under the legal basis of explicit consent or if the data was shared on other GDPR grounds. The third node states which form of de-identification is used. The lower section shows four sets of bar charts: Security, Ease of use, Sustainability, and Implementability. For each set, all four options (A, B, C and D) are rated discretely for that topic from 1 to 5 by the authors. The ratings are relative, and the scale is explained in the legend at the bottom of the figure. Ratings are subjective; therefore, interpretations can differ. This figure shows that approach A allows the user to download pseudonymised data of patients who provided consent. Approach B also allows the user to download the data yet does not require consent, thus requiring another legal basis under the GDPR and pseudonymises the data. Approach C is identical to B, except it anonymises the data. Finally, approach D incorporates cloud computing, meaning that the users cannot download the data but must access it through an online portal containing pseudonymised patient data without the required consent. All four approaches are legally and practically valid but have different implications on data security, ease of use, sustainability, and implementability.

**Fig. 3:**
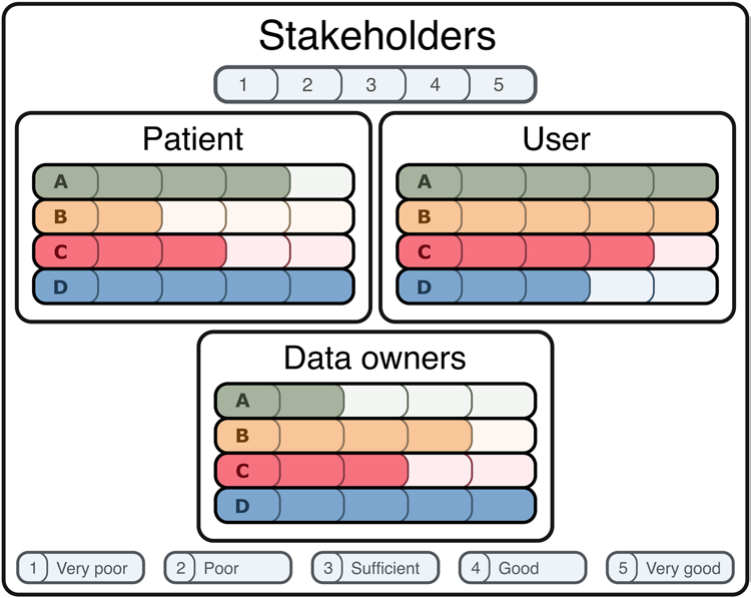
Implications of the four different approaches presented in Fig. 2 for the different stakeholders: the patient, user, and data owner. The more a bar is filled, the better the approach is for the stakeholder. Ratings are subjective; therefore, interpretations can differ. The patient benefits most from approach D, as cloud computing is the most secure option, minimising the risk of a patient’s sensitive data being leaked. Also, it is the most transparent, as all activities related to the data can be monitored. Furthermore, its sustainability makes it ideal for users. However, not only the quality of the data is essential for the user, but also its usability. Therefore, users might prefer approaches A and B since these are easy to use and relatively sustainable, data quality of approach C can be worse since it is fully anonymous and cannot easily be enriched, making it less useful for the user. The data owners are responsible for building and maintaining the database. Consequently, approach A is the least preferred option, as consent can be challenging and time-consuming to implement. Approach C might also not be preferred because the anonymisation process can be difficult and database updates even harder. Approach B is already much better for the data owners due to its sustainability; however, it can be hard to implement legally. Although approach D is arguably the most challenging to implement for the data owner, we still expect this is the preferred option as it offers the most control over the database and its use. Furthermore, the cloud computing infrastructure can be used for many different data sets, meaning that it only has to be set up once and can be maintained for all shared data sets.

Based on the four successful open ICU databases and rules ordained in the GDPR, we present four possible approaches for openly sharing sensitive healthcare data in Fig. 2. The four approaches have different implications on data security, ease of use, sustainability, and implementability. We argue that approach A, using patient consent as a legal basis and pseudonymisation as a de-identification strategy, is one of the least secure approaches as de-identified patient data can be accessed and redistributed, while patient re-identification is possible with the re-identification keys. It is also difficult to implement, as asking for consent can be time-consuming and virtually impossible retrospectively. However, it is easy to use and sustainable since data is easily accessible and updateable, given the possibility of patient re-identification. Approach B, pseudonymising patient data without consent, is comparable to approach A, but can be easier to implement since asking for patient consent is not required. However, another valid legal basis is required to share personal data, potentially requiring stricter governance. Approach C does not require consent and anonymises the data. This makes approach C the least sustainable, as the database must be rebuilt each time new variables are added, as patients cannot be re-identified. However, implementation is easy as this requires the least governance because the data is already secure, given that patients cannot be re-identified. This also makes the data easy to use, and commercial parties often prefer anonymised data as this minimises their legal risk. Approach D is not based on the four databases but suggests that future databases host pseudonymised data in the cloud without consent, making it the most secure approach, in our opinion. In addition, the institutes can audit user behaviour to prevent malpractice and the data from being redistributed by its users as it cannot be downloaded locally. Due to the complexity of cloud infrastructures, it might be more challenging to implement and not the most straightforward to use. Nevertheless, we expect this to improve rapidly over the upcoming years as the technology for cloud services gains momentum. The four approaches also have different implications for the stakeholders, which are visualised in Fig. 3.

### Limitations

This study has limitations, as we were unaware of any failed European open ICU data projects. Although we could have learned from failed projects, we feel that the four successful projects provide essential leads to further open European ICU data. Furthermore, we did neither perform a formal qualitative interview method nor interview all team members with different expertise in publishing these four open European ICU databases. From each database team, we interviewed people with varying backgrounds, which may produce bias in the questionnaire answers. Nevertheless, we also based our investigation on published results of opening these European ICU databases and, in that way, covered more expertise involved in opening these datasets. We could only collect minimal information on the legal bases used by the databases to share their data under the GDPR legally. For example, we only know that AmsterdamUMCdb used article 89, which is in accordance with article 9 (2j). HiRID might have taken a similar approach as it is a more general ICU database, whereas Sanitas and HM Hospitales could have used alternative valid legal bases during the COVID-19 pandemic^6^. Also, preferences regarding the approaches to sharing open healthcare data (Fig. 2) are subjective and can thus differ per individual. Nevertheless, they still provide insight into the potential consequences of decisions made when publishing healthcare data and are intended to elicit thought and discussion.

### Recommendations and conclusion

**Fig. 4:**
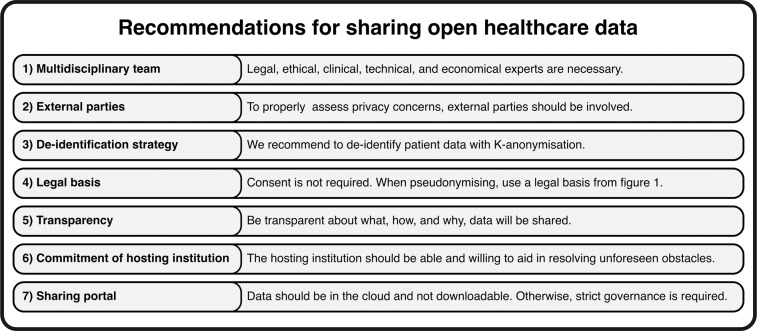
Recommendations for sharing open healthcare data under the General Data Protection Regulation.

We have several recommendations for sharing sensitive healthcare data within the EU (Fig. 4). First, it is important to have a multidisciplinary team of experts with in-house knowledge to tackle legal, ethical, economic, and technical issues. Second, external parties can be involved in assessing the privacy and data protection risks to acquire unbiased risk assessments. Third, the risk of re-identification (for both anonymised and pseudonymised data) should be adequately accounted for. The de-identification methodology we recommend is K-anonymisation, as performed by AmsterdamUMCdb. Fourth, patient consent is not always required by the GDPR, as indicated in Fig. 1. Fifth, adherence to transparency when publishing open data and trust between patients and other stakeholders is crucial when sharing health data^30,31^. This includes the legal obligation for the hosting institution to inform patients through its privacy statement that their data can be shared and reused for investigation purposes. Sixth, the commitment of the hosting institution is required for a successful publication process. The institution should be willing to back up the project when unforeseen obstacles present themselves (i.e., costs and/or effort). Ultimately, data should be stored and analysed in the cloud, if possible, so the data cannot be downloaded. If infeasible, strict governance should be implemented. To guide future initiatives in sharing open healthcare data, we included a Gantt chart in the supplementary information^14^ as a planning guideline. In conclusion, publishing open health data in the EU might be challenging, but it is essential for developing modern-day healthcare. The experiences and lessons learned from these four successful databases can guide the development of new open European ICU databases.

## Methods

Four published open European ICU database projects were selected and interrogated: AmsterdamUMCdb^32^, HiRID^33^, Sanitas^34^, and HM^35^. A committee of experts, including intensivists, anaesthesiologists, statisticians, epidemiologists, and data scientists, was assembled to generate a questionnaire with relevant questions on the main aspects of the database construction process. A methodology inspired by the Delphi method^36^ was adopted to reach a consensus on the questions iteratively. The questions were intended to capture how those centres approached publishing an open healthcare database and the steps to succeed in such an endeavour, including the funding, legal, ethical, anonymisation, and governance aspects. All four centres filled out the initial questionnaire. Based on the initial response, the expert committee constructed a second version containing some additional and more specific questions. With the second version, a remote interview was set up with each database to obtain nuances and answers to the new questions not covered in the first version (see Questionnaires section in Supplementary^14^ information). The profile of interviewees included clinicians with profound knowledge of data science, researchers, project managers, data protection and governance officers, and heads of legal and IT departments. After transcription of the responses, each transcript was sent to each centre of origin for final approval. The answers can be found in Supplementary Table S1^14^ and are summarised in Table 1. The manuscript was reviewed by data privacy experts/lawyers to ensure the correct formulation of the legal principles.

## Data Availability

The questionnaires and complete answers from the four databases are available in the supplementary information^14^, which can be accessed via the following 10.6084/m9.figshare.22643419. No other data was used for this paper.

## Code availability

No code was written or used for this paper.
